# IL-4 type 1 receptor signaling up-regulates *KCNN4* expression, and increases the KCa3.1 current and its contribution to migration of alternative-activated microglia

**DOI:** 10.3389/fncel.2014.00183

**Published:** 2014-07-01

**Authors:** Roger Ferreira, Starlee Lively, Lyanne C. Schlichter

**Affiliations:** ^1^Genes and Development Division, Toronto Western Research Institute, University Health NetworkToronto, ON, Canada; ^2^Department of Physiology, University of TorontoToronto, ON, Canada

**Keywords:** alternative microglial activation, AP-1 transcription factor, KCa3.1/SK4 channel, IL-4 signaling, M2 macrophage activation, microglial migration, Ras/MEK/ERK signaling, type I IL-4 receptor

## Abstract

The Ca^2+^-activated K^+^ channel, KCa3.1 (*KCNN4*/IK1/SK4), contributes to “classical,” pro-inflammatory activation of microglia, and KCa3.1 blockers have improved the outcome in several rodent models of CNS damage. For instance, blocking KCa3.1 with TRAM-34 rescued retinal ganglion neurons after optic nerve damage *in vivo* and, reduced p38 MAP kinase activation, production of reactive oxygen and nitrogen species, and neurotoxicity by microglia *in vitro*. In pursuing the therapeutic potential of KCa3.1 blockers, it is crucial to assess KCa3.1 contributions to other microglial functions and activation states, especially the IL-4-induced “alternative” activation state that can counteract pro-inflammatory states. We recently found that IL-4 increases microglia migration – a crucial function in the healthy and damaged CNS – and that KCa3.1 contributes to P_2_Y_2_ receptor-stimulated migration. Here, we discovered that KCa3.1 is greatly increased in alternative-activated rat microglia and then contributes to an enhanced migratory capacity. IL-4 up-regulated *KCNN4* mRNA (by 6 h) and greatly increased the KCa3.1 current by 1 day, and this required *de novo* protein synthesis. The increase in current was sustained for at least 6 days. IL-4 increased microglial migration and this was reversed by blocking KCa3.1 with TRAM-34. A panel of inhibitors of signal-transduction mediators was used to analyze contributions of IL-4-related signaling pathways. Induction of *KCNN4* mRNA and KCa3.1 current was mediated specifically through IL-4 binding to the type I receptor and, surprisingly, it required JAK3, Ras/MEK/ERK signaling and the transcription factor, activator protein-1, rather than JAK2, STAT6, or phosphatidylinositol 3-kinase.The same receptor subtype and pathway were required for the enhanced KCa3.1-dependent migration. In providing the first direct signaling link between an IL-4 receptor, expression and roles of an ion channel, this study also highlights the potential importance of KCa3.1 in alternative-activated microglia.

## INTRODUCTION

Following CNS injury, persistent inflammation can promote secondary tissue injury through excess production of reactive oxygen and nitrogen species, cytokines, metalloproteases, and other mediators. Thus, it is important to limit the magnitude and duration of the innate inflammatory response. *In vitro* studies of macrophages, and more recently of microglia, show that IL-4 polarizes them to an “alternative” activation state (or “M2”), while IL-10 and TGFβ help resolve pro-inflammatory, “classical” activation (“M1”; Gordon, 2003; Colton, 2009; Varin and Gordon, 2009; Van Dyken and Locksley, 2013). IL-4 binds to the IL-4 receptor α chain (IL-4Rα) on type I and II receptors (Nelms et al., 1999; Van Dyken and Locksley, 2013). The type II receptor can use IL-4 and IL-13; whereas, the type I receptor uses IL-4 only, and it induces larger changes in gene expression (Heller et al., 2008). Both receptors initiate signaling cascades that alter gene expression and cell behavior but the pathways differ. Type I receptors signal through signal transducer and activator of transcription 6 (STAT6) and insulin receptor substrate 2 (IRS2), while type II receptors only signal through STAT6 (Sica and Mantovani, 2012; Van Dyken and Locksley, 2013).

In a microarray analysis of IL-4 treated human macrophages (Pello et al., 2012), we noted that *KCNN4* mRNA was increased. This was surprising because *KCNN4* encodes the Ca^2+^-activated K^+^ channel, KCa3.1 (IK1/SK4; Joiner et al., 1997; Khanna et al., 1999), which we found is involved in several functions of classical-activated rat microglia. That is, KCa3.1 blockers inhibited the respiratory burst (Khanna et al., 2001), and LPS-induced p38 MAPK activation, NO production, and neurotoxicity (Kaushal et al., 2007). In the latter study, LPS did not affect *KCNN4* mRNA expression at 24 h but the KCa3.1 current was not examined. Several *in vivo* studies using the selective KCa3.1 blocker, TRAM-34, show improved outcomes in rodent models of CNS conditions with prominent inflammation; i.e., models of multiple sclerosis (Reich et al., 2005), optic nerve damage (Kaushal et al., 2007), spinal cord injury (Bouhy et al., 2011), and ischemic stroke (Chen et al., 2011). Because KCa3.1 is now considered a therapeutic target for reducing the pro-inflammatory state of the injured CNS (Wulff and Zhorov, 2008; Skaper, 2011; Maezawa et al., 2012), it is essential to determine its roles in other microglial activation states and cell functions. One important microglial function is migration to the damage site. We recently reported that blocking KCa3.1 with TRAM-34 inhibits chemotactic migration of rat microglia following P_2_Y_2_ purinergic receptor stimulation (Ferreira and Schlichter, 2013), and that IL-4-induced alternative activation increases the microglial migratory capacity and range of enzymes used for matrix degradation (Lively and Schlichter, 2013).

Therefore, we first asked whether IL-4 up-regulates expression of *KCNN4* and the KCa3.1 current in rat microglia. Having found this to be the case, we analyzed contributions of several effector molecules downstream of the two subtypes of IL-4 receptor: JAK2, JAK3, STAT6, phosphatidylinositol 3-kinase (PI3K), MEK, and the transcription factor, AP1. Finally, we assessed the role of KCa3.1 and these signaling pathways in the increased migratory capacity of IL-4-treated microglia. Together, our results indicate that the type I IL-4 receptor, Ras/MEK/ERK pathway, and activator protein-1 (AP-1) are responsible for increasing *KCNN4* expression, KCa3.1 current, and KCa3.1-dependent migratory capacity.

## MATERIALS AND METHODS

### PRIMARY RAT MICROGLIA CULTURES

All procedures on animals were in accordance with guidelines from the Canadian Council on Animal Care and approved by the University Health Network Animal Care Committee. Microglia were isolated from 1 to 2 day-old Sprague–Dawley rat pups (Charles River, St. Constant, PQ, Canada) using our standard protocols, which yield ≥99% microglia with little or no spontaneous activation (Sivagnanam et al., 2010; Liu et al., 2013; Lively and Schlichter, 2013; present study). Briefly, after the meninges were removed, the whole brain was minced, centrifuged (300 × *g*, 10 min), re-suspended in Minimal Essential Medium (MEM; Invitrogen, Carlsbad, CA, USA) supplemented with 10% fetal bovine serum (FBS; Wisent, St-Bruno, PQ, Canada) and 0.05 mg/ml gentamycin (Invitrogen), and seeded in tissue culture flasks. Cells were then cultured at 37°C and 5% CO_2_ for 48 h, washed and cultured for an additional 5–6 days. To separate the microglia from the bed of astrocytes, the flasks were shaken for 3–4 h (65 rpm, 37°C, 5% CO_2_), and the supernatant was centrifuged (300 × *g*, 10 min) to spin down the microglia, which were then re-suspended in MEM supplemented with 2% FBS, and plated at densities appropriate for each assay. For mRNA analysis and electrophysiology, microglia were seeded at 1–2 million cells/35 mm culture dish and 75,000 cells/coverslip, respectively, then grown for 1–2 days before treatment with IL-4 or inhibitors. Similarly, for the proliferation assay, cells were seeded at 10,000 cells/well of a 96-well plate and grown for 18 h prior to treatments. For the migration assay, microglia were seeded (30,000 cells/well) onto the inner wells of Transwell^TM^ chambers and allowed to settle for 1 h before treatment.

### CHEMICALS

To induce alternative activation, microglia were treated with rat recombinant IL-4 (R&D Systems Inc., Minneapolis, MN, USA), as before (Liu et al., 2013; Lively and Schlichter, 2013). The JAK2/3 inhibitor, AG490 (EMD Millipore, Toronto, ON, Canada) was used at 10 μM, a concentration previously shown to inhibit JAK signaling in primary microglia (Kim et al., 2002; Huang et al., 2008). The JAK2-selective inhibitor, TG101348 (Selleckchem, Houston, TX, USA; IC_50_ = 3 nM) and JAK 3-selective inhibitor, tofacitinib (Selleckchem; IC_50_ = 1 nM) were used at 30 nM and 10 nM, respectively. STAT6 was inhibited using 200 nM AS1517499 (IC_50_ = 21 nM; Axon Medchem BV, Groningen, Netherlands). 100 nM wortmannin (EMD Millipore; IC_50_ = 5 nM) was used to inhibit PI3K activity. The mitogen-activated protein kinase kinase (MAPKK, also known as MEK) inhibitors, U0126 (IC_50_ = 72 nM for MEK1, 58 nM for MEK2) and PD098059 (IC_50_ = 2–7 μM) were obtained from Sigma–Aldrich (Oakville, ON, Canada), and used at 10 μM and 20 μM, respectively. AP-1 is a c-Fos/c-Jun heterodimer that can bind to the *KCNN4* promoter and initiate transcription in activated T lymphocytes (Ghanshani et al., 2000). To inhibit AP-1, the retinoid, SR11302 (R&D Systems) was used at 1 μM; a concentration shown to inhibit AP-1 activity (Hu et al., 2009). To inhibit protein synthesis, cycloheximide (CHX; Sigma) was used at 10 nM, a concentration that is effective in primary microglia (Chen et al., 2009). TRAM-34 (Sigma) was used at 1 μM to selectively block KCa3.1 (IC_50_ = 25 nM; Wulff et al., 2000). All inhibitors were diluted in DMSO. None of the compounds were toxic to rat microglia at the concentrations used. The KCa channel activators, riluzole, 1-EBIO, and NS309 (all from Sigma), were used at 300 μM, 300 μM, and 500 nM, respectively.

### MULTIPLEXED GENE EXPRESSION ANALYSIS (NanoString nCounter^TM^)

This high-throughput method has similar sensitivity to real-time qRT-PCR but can analyze expression of many genes using a single RNA sample (Geiss et al., 2008). Total RNA was extracted as previously described (Sivagnanam et al., 2010; Liu et al., 2013; Lively and Schlichter, 2013) using TRIzol reagent (Invitrogen), followed by RNeasy Mini Kit (QIAGEN, Mississauga, ON, Canada) for further purification. RNA samples were stored at –80°C. Genes analyzed in this study were chosen based on previous reports of their increased expression following IL-4 treatment (either from our lab or reported in the literature) as well as genes of special relevance to this study (e.g., *KCNN4*). Each gene was recognized by a probe set that was designed and synthesized by NanoString nCounter^TM^ technologies (**Table 1**). A probe set consists of capture and reporter probes, which are complimentary sequences of 35–50 base pairs that are designed to bind specifically to the mRNA of interest. The capture probe also contains a short sequence linked to biotin, while the reporter probe is coupled to a unique color-coded tag used for detection.

**Table 1:** Target sequences used to design probe sets for multiplexed gene expression analysis (NanoString nCounter^TM^).

Gene	Genbank Accession #	Target sequence
CD163	NM_001107887.1	AGTTTCCTCAAGAGGAGAGGTCTTGATACATCAAGTTCAGTACCAAGAGATGGATTCGAAGACGGATGATCTGGACTTGCTGAAATCCTCGGGTTGGCAT
HPRT1	NM_012583.2	AGCTTCCTCCTCAGACCGCTTTTCCCGCGAGCCGACCGGTTCTGTCATGTCGACCCTCAGTCCCAGCGTCGTGATTAGTGATGATGAACCAGGTTATGAC
IL-4Rα	NM_133380.2	GGGTGTCAGCATCTCCTGCATCTGCATCCTATTGTTTTGCCTGACCTGTTACTTCAGCATTATCAAGATTAAGAAGATATGGTGGGACCAGATTCCCACT
*KCNN4*	NM_023021.1	ATCGGACTCATGGTGCTGCACGCTGAGATGTTGTGGTTCCTGGGTTGCAAGTGGGTGCTGTACCTGCTCTTGGTTAAGTGTTTAATCACGCTGTCCACTG
MRC1	NM_001106123.1	CTTTGGAATCAAGGGCACAGAGCTATATTTTAACTATGGCAACAGGCAAGAAAAGAATATCAAGCTTTACAAAGGTTCCGGTTTGTGGAGCAGATGGAAG
STAT6	NM_001044250.1	GTGGTTTGATGGTGTCCTGGACCTCACTAAACGCTGTCTTCGGAGCTACTGGTCAGATCGGCTGATCATCGGCTTTATCAGTAAGCAATATGTCACTAGC


For both IL-4-treated microglia and corresponding control (unstimulated) microglia, samples were harvested from separate cultures isolated from individual rat pups (*n* = 5 pups). We then supplied 200 ng of extracted RNA from unstimulated and IL-4-treated cultures to the Princess Margaret Genomics Centre, Toronto, Canada^1^, which conducted the NanoString nCounter^TM^ analysis. Prior to running the analysis, samples were assessed for purity using Nanodrop 1000. Data were normalized to expression of the housekeeping gene, hypoxanthine guanine phosphoribosyl transferase (*HPRT1*),which we find to be especially stable in primary rat microglia under all treatments we have investigated (Sivagnanam et al., 2010; Liu et al., 2013; Lively and Schlichter, 2013). Sample preparation, hybridization, detection, and scanning were executed following NanoString Technologies’ recommendations. mRNA transcripts were analyzed and quantified using the nCounter^TM^ digital analyzer software^2^.

### QUANTITATIVE REAL-TIME REVERSE-TRANSCRIPTASE POLYMERASE CHAIN REACTION (qRT-PCR)

RNA was extracted as described above. The following primers for *KCNN4* and the housekeeping gene, *HPRT1*, were designed using “Primer3Output”^3^. *KCNN4:* forward (5′-GCTGGAGCAGGAGAAGAGG-3′) and reverse (5′-AAAGGAGGAAGGCAGTGGA-3′). *HPRT1*: forward (5′-CAGTACAGCCCCAAAATGGT-3′) and reverse (5′-CAAGGGC-ATATCCAACAACA-3′). cDNA was first synthesized by reverse transcription according to the manufacturer’s instructions (Invitrogen). In brief, 0.8 μg of total RNA was reverse transcribed in 20 μl volume using 200 U of SuperScriptII RNase reverse transcriptase, with 0.5 mM dNTPs and 0.5 μM oligo dT (Invitrogen). Using an ABI PRISM 7700 Sequence Detection System (PE Biosystems, Foster City, CA, USA), amplification was then performed as follows: (1) 50°C for 2 min, (2) 95°C for 10 min, (3) 40 cycles at 95°C for 15 s and 60°C for 60 s, and (4) a dissociation step (95°C for 15 s, 60°C for 15 s, 95°C for 15 s). “No-template” and “no-amplification” controls were included for each gene. Specific amplification was confirmed by the single peak on melt curves. The threshold cycle (CT) for *KCNN4* was normalized to that of *HPRT1*.

### MICROGLIA STAINING

Rat microglia were seeded at ~6 × 10^4^ cells/15 mm diameter coverslip, cultured for 1 day in 2% FBS, and then stimulated with 20 ng/ml rat recombinant IL-4. Cells were fixed 24 h later in 4% paraformaldehyde (Electron Microscopy Sciences, Hatfield, PA, USA) at room temperature for 10 min and then permeabilized with 0.2% Triton X-100 for 5 min. To examine morphology as a function of activation state, microglia were stained with FITC-conjugated tomato lectin (TL; 1:500, 15 min; Sigma) and counterstained with the nuclear dye, 4′,6-diamidino-2-phenylindole (DAPI; 1:3000 in PBS, 5 min; Invitrogen).

### PATCH-CLAMP ELECTROPHYSIOLOGY

Primary rat microglia were plated on 15 mm diameter coverslips (~7.5 × 10^4^/coverslip), and mounted in a model RC-25 perfusion chamber (Warner Instruments, Hamden, CT) for patch clamp recordings. The cells were superfused with an extracellular (bath) solution containing (in mM): 125 NaCl, 5 KCl, 1 MgCl_2_, 1 CaCl_2_, 5 glucose, and 10 HEPES, adjusted to pH 7.4 (with NaOH) and to ~300 mOsm with sucrose. Bath solutions were exchanged using a gravity-driven perfusion system flowing at 1.5–2 ml/min and all recordings were made at room temperature. Whole-cell recordings were made with pipettes pulled from thin-walled borosilicate glass (WPI, Sarasota, FL) using a Narishige puller (Narishige Scientific, Setagaya-Ku, Tokyo) to a resistance of 6-9 MΩ, which provided good seal stability. Pipettes were filled with a solution containing (in mM): 100 K-aspartate, 40 KCl, 1 MgCl_2_, 2 MgATP, 5 EGTA, 4.3 CaCl_2_, 10 HEPES, pH adjusted to 7.2 with KOH, 280 mOsm/kg H_2_O. This intracellular solution had 1.0 μM free Ca^2+^, as calculated with WEBMAXC Extended software^4^, which was expected to facilitate KCa3.1 channel activation. However, as described in the Results, it was necessary to also add a KCa channel activator. Recordings were made with an Axon Multiclamp 700A amplifier (Molecular Devices, Sunnyvale, CA, USA), and compensated on-line to minimize the capacitance transient, which can be large due to the high membrane resistance, up to several gigaohms (Newell and Schlichter, 2005). Patch-clamp data were filtered at 5 kHz, and acquired and digitized using a Digidata 1322A board with pClamp software (Molecular Devices, Sunnyvale, CA, USA). The junction potential was reduced by using agar bridges made with bath solution, and was about –5 mV, as calculated using the utility in pClamp.

### MIGRATION ASSAY

Microglia were seeded on filters with 8 μm-diameter pores placed in Transwell^TM^ chambers (VWR). After 30 min, MEM supplemented with 2% FBS was added to the upper and lower wells. After 1 h, microglia were left unstimulated (controls) or incubated with 20 ng/ml IL-4, with or without one of the following compounds: 1 μM TRAM-34, 10 nM cycloheximide, 10 μM AG490, 10 nM tofacitinib, 30 nM TG101348, 200 nM AS1517499, 100 nM wortmannin, 20 μM PD098059, 10 μM U0126, or 1 μM SR11302. After incubating for 24 h (37°C, 5% CO_2_), microglia on the filters were fixed in 4% paraformaldehyde for 10 min and washed with PBS. To remove the remaining cells that had not migrated through the filter, the upper side of the filter was swirled with a Q-tip. Cells that migrated to the underside of the filters were visualized by adding 0.3% crystal violet for 1 min, followed by a quick wash in PBS to remove free dye. Migrated cells were counted (five random fields/filter) at 20× magnification using an Olympus CK2 inverted microscope (Olympus, Tokyo, Japan). For each culture, total cell counts obtained from experimental Transwell chambers (IL-4 treated ± an antagonist) were normalized to the total cell counts from corresponding unstimulated (control) Transwells.

### CELL PROLIFERATION ASSAY

The CyQUANT NF cell proliferation assay (Invitrogen) was used to measure cell proliferation. Microglia were seeded at 10^3^/well on a 96-well flat-bottom plate. To generate a standard curve of fluorescence intensity versus cell number, we added a range of 0–30,000 cells in separate wells. Cells were incubated overnight (37°C, 5% CO_2_) in MEM supplemented with 2% FBS. The following morning, 20 ng/ml IL-4, 1 μM TRAM-34 or both were added to test wells, incubated for a further 24 h, and then the CyQUANT assay was performed according to the manufacturer’s protocol. In brief, after 30 min incubation in the dye-binding solution (37°C, 5% CO_2_), fluorescence intensity was measured using a multi-label plate counter (Victor^3^ 1420, Perkin Elmer, Woodbridge, ON, Canada). Excitation was set at 485 nm and emission was measured at 535 nm, with 0.1 s readings at 3 mm from the bottom of the plate taken in triplicate. For treatment samples, cell numbers were calculated by interpolation from the standard curve.

### STATISTICAL ANALYSIS

All graphical data are expressed as mean ± SEM. Changes in gene expression from NanoString were analyzed using a 2-way ANOVA with Bonferroni’s *post hoc* test; the two independent variables were time and stimulation (untreated versus IL-4 treated). For analyzing migration and proliferation data, a 2-way ANOVA followed by Bonferroni’s test was used to determine the effects of stimulation and multiple inhibitors. When only one variable was involved, Student’s unpaired *t*-test was used; i.e., when assessing effects of stimulation alone (untreated versus IL-4 treated) on KCa3.1 current amplitude or *KCNN4* mRNA expression (qRT-PCR) at a single time. When analyzing effects of multiple inhibitors on a single outcome (KCa3.1 current, *KCNN4* mRNA, migration), a 1-way ANOVA with Tukey’s *post hoc* test was used. All analyses were conducted using GraphPad Prism ver 5.01 (San Diego, CA, USA). Values of *p* < 0.05 were taken as statistically significant.

## RESULTS

### IL-4-INDUCED ALTERNATIVE ACTIVATION EVOKES PROLONGED UP-REGULATION OF *KCNN4* EXPRESSION AND KCa3.1 CURRENT

#### mRNA expression

**FIGURE 1:**
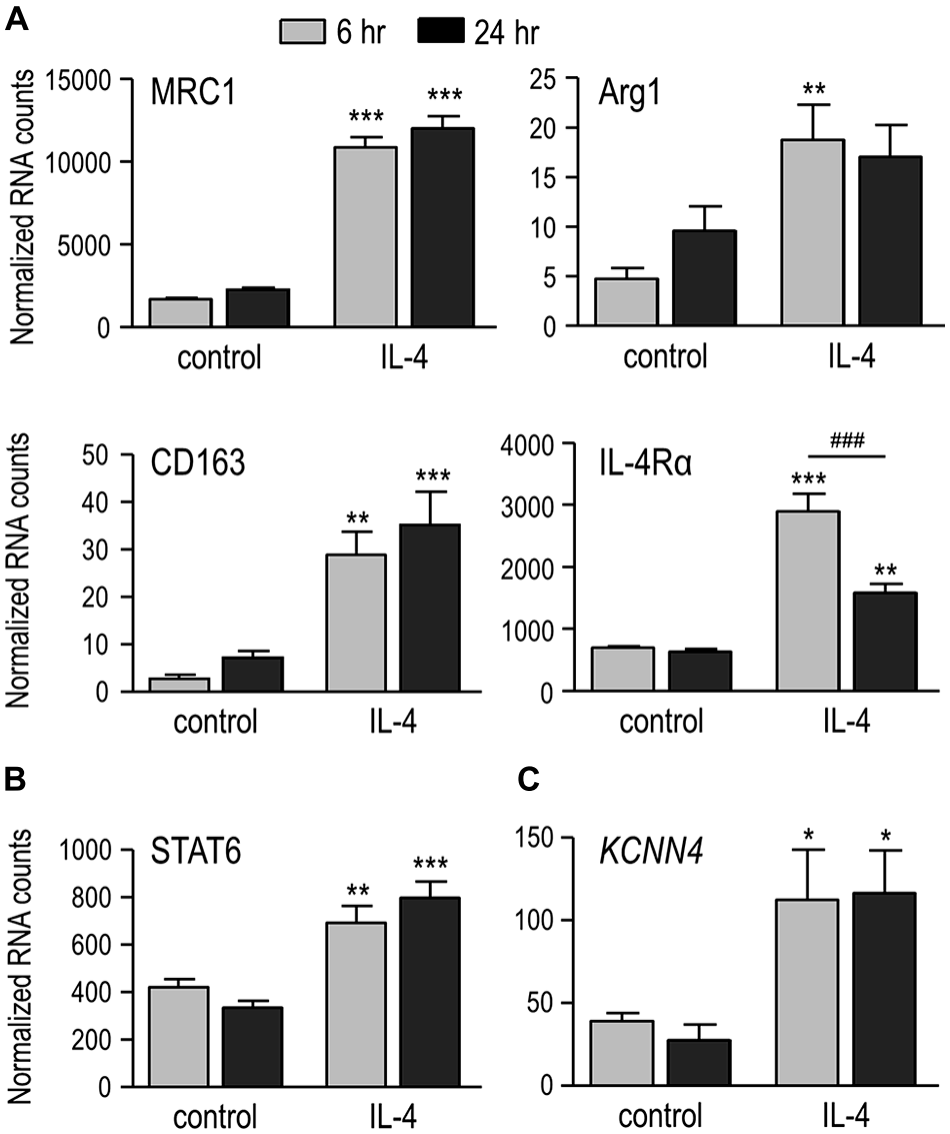
**IL-4 treatment induces alternative activation, and up-regulates IL-4 signaling genes and *KCNN4* mRNA. (A)** Treating rat microglia with rat recombinant IL-4 (20 ng/ml; 6 or 24 h) up-regulated expression of “alternative” activation markers: C-type mannose receptor 1 (MRC1), IL-4 receptor α subunit (IL-4Rα), CD163 and arginase 1 (Arg1). **(B,C)** Also up-regulated were “signal transducer and activator of transcription 6” (STAT6), a transcription factor involved in the IL-4 signaling pathway; and the Ca^2+^-activated K^+^ channel, *KCNN4*. Gene expression was analyzed using NanoString and normalized to the housekeeping gene, *HPRT1*. Values are expressed as mean ± SEM for five replicates from different cell cultures. Two-way ANOVA with Bonferroni’s *post hoc* test revealed differences between unstimulated and IL-4-treated microglia (*) and some time-dependent changes (#). One symbol indicates *p* < 0.05, two symbols, *p* < 0.01, three symbols, *p* < 0.001.

It is well known that IL-4 evokes alternative activation of microglia (Colton et al., 2006; Colton, 2009), as we recently showed for rat microglia at 24 h after IL-4 treatment (Liu et al., 2013; Lively and Schlichter, 2013). Here, we show increased expression of several prototypical alternative activation genes as early as 6 h after treatment with 20 ng/ml of rat recombinant IL-4 (**Figure 1A**). Compared with time-matched unstimulated microglia, the increases at 6 and 24 h were 6.5- and 5.3-fold for the C-type mannose receptor 1 (MRC1), 4.2- and 2.5-fold for the IL-4Rα, 3.9- and 1.8-fold for arginase 1 (Arg1), and 10.8- and 5.0-fold for CD163. In addition, STAT6 – a primary transcription factor downstream of IL-4 receptors (reviewed in Sica and Mantovani, 2012) – was increased by 1.6- and 2.4-fold at 6 and 24 h, respectively (**Figure 1B**). IL-4 treatment also increased *KCNN4*, which encodes the intermediate conductance, Ca^2+^-activated K^+^ channel, KCa3.1 (see Introduction), by 2.9-fold at 6 h and 4.2-fold at 24 h (**Figure 1C**).

#### KCa3.1 Current

Whole-cell currents were compared between unstimulated microglia and cells treated for 24 h with IL-4. The KCa3.1 current was quantified as the component blocked by 1 μM TRAM-34 (Wulff et al., 2000), a procedure that eliminated the inward-rectifier (Kir2.1) current that is prevalent at negative membrane potentials and the depolarization-activated outward Kv1.3 current (Schlichter et al., 1996; Kotecha and Schlichter, 1999; Fordyce et al., 2005; Newell and Schlichter, 2005). For each cell, repeated voltage ramps were applied to examine current-versus-voltage (I–V) relations, and to quantify the current amplitude and prevalence (proportion of cells expressing the current).

**FIGURE 2:**
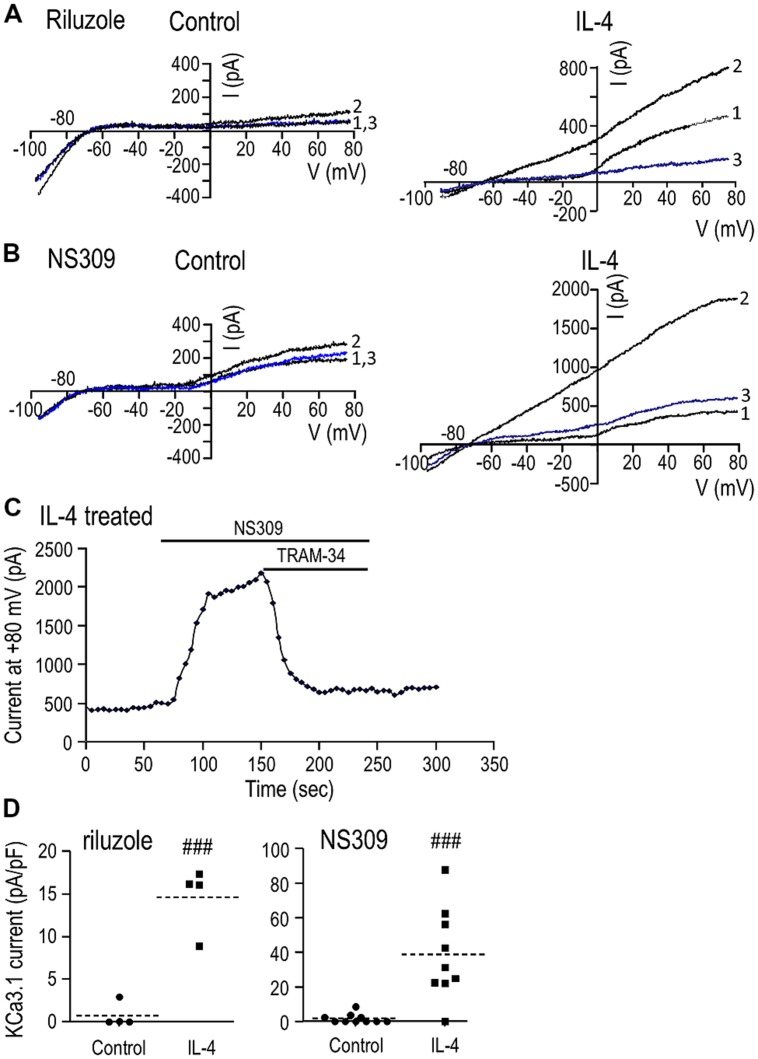
**IL-4 treatment induces a robust KCa3.1 current. (A,B)** Representative currents in response to repeated voltage ramps from –100 to +80 mV from a holding potential of –70 mV. Unstimulated microglia (left panels) were compared with cells treated for 24 h with 20 ng/ml of IL-4 (right panels). Each example shows superimposed currents in normal bath solution (traces labeled “1”), after adding a KCa channel activator (300 μM riluzole or 500 nM NS309; traces labeled “2”), and after adding the selective KCa3.1 blocker, 1 μM TRAM-34, in the continued presence of the channel activator (blue traces labeled “3”). **(C)** An example of the time course of current activation by NS309 (measured at +80 mV; same IL-4-treated cell as **B**) and block by 1 μM TRAM-34. **(D)** A scatterplot of the TRAM-34-sensitive KCa3.1 component in each unstimulated and IL-4-treated microglial cell summarizes the KCa3.1 current density (pA/pF) and prevalence (proportion of cells expressing KCa3.1 current). Dashed lines indicate mean current densities (note differences in the Y-axis scales). Statistical differences between control (unstimulated) and IL-4-treated microglia were determined by an unpaired *t*-test for each of the activators: ^###^*p* < 0.001.

(i) Unstimulated microglia. Attempts to activate the current by simply elevating intracellular (pipette) Ca^2+^ to 1 μM were unsuccessful (>50 cells tested), despite allowing the cytosol to equilibrate with the pipette solution for up to 10 min (~10 cells). This lack of current activation is consistent with our recent study of the MLS-9 microglia cell line using pipettes of a similar resistance (4-7 MΩ), in which a KCa3.1 current was activated by 1 μM Ca^2+^ in only 1/10 cells (Liu et al., 2013). We found that the pipette solution diffuses into microglial cells within 1 min after break-in, as judged by the reporter dye, Ca-Green (molecular weight: 1.14 kDa). Next, we bath applied three well-established KCa channel activators: riluzole (300 μM), which reliably activated KCa3.1 in MLS-9 cells (Liu et al., 2013), 1-EBIO (300 μM), and its more potent derivative, NS309 (500 nM; Wulff et al., 2007). Elevated intracellular Ca^2+^alone did not activate a current (0/19 cells; example traces labeled “1” in **Figures 2A,B**). The KCa activators evoked little or no current (traces labeled “2”). A small current (<100 pA) was seen in only 1/4 cells with riluzole (**Figure 2A**), 3/10 cells with NS309 (**Figure 2B**; example showing the largest responder), and 1/5 cells with 1-EBIO (not illustrated). Not surprisingly, TRAM-34 had little or no effect in control microglia (traces labeled “3”).

(ii) IL-4-treated microglia. With 1 μM intracellular Ca^2+^ alone (traces labeled “1”), there was very little current between –80 and –20 mV, but a variable-amplitude depolarization-activated Kv1.3 current was often seen as an inflection in the I–V relation. [The Kv1.3 current showed considerable variability (even in control cells; compare **Figures 2A,B**), and the effects of microglial activation state will be examined in a future study.] Most importantly for the present study, all three KCa activators evoked large currents (traces labeled “2”). As is diagnostic of KCa3.1, the current was present at all voltages tested (-100 to +80 mV), reversed near the K^+^ Nernst potential, and was substantially blocked (average of 95%, *n* = 14) by 1 μM TRAM-34 (traces labeled “3”). [The current with riluzole+TRAM-34 (**Figure 2A**, trace “3”) was smaller than the control (trace “1”) because riluzole also inhibits Kv1.3 (Bellingham, 2011).] An example of the time course of current activation by NS309 and block by TRAM-34 is shown in **Figure 2C**. KCa3.1 currents were evoked by riluzole in 4/4 cells, by NS309 in 8/9 cells, and by 1-EBIO in 5/5 cells (not illustrated). The mean amplitude of the TRAM-34-sensitive current (i.e., trace 2 minus trace 3) was compared between unstimulated and IL-4-treated microglia (**Figure 2D**). Riluzole-evoked KCa3.1 currents were 0.7 ± 0.7 pA/pF in unstimulated microglia versus 14.6 ± 1.9 pA/pF in IL-4-treated cells (*n* = 4 each). NS309-evoked currents were 1.7 ± 0.9 pA/pF in unstimulated (*n* = 10) versus 38.9 ± 8.8 pA/pF in IL-4-treated microglia (*n* = 9). 1-EBIO-evoked currents were 0.8 ± 0.8 pA/pF in unstimulated versus 22.9 ± 5.4 pA/pF in IL-4-treated cells (*n* = 5 each; data not illustrated). [A similar current was evoked by the activators in >50 other IL-4-treated microglia, but TRAM-34 was not added to quantify the KCa3.1 component.] Riluzole, NS309, and 1-EBIO are known to also activate KCa2.3 channels with approximately five-fold lower potency (Wulff and Zhorov, 2008); and although not investigated further in the present study, a small KCa current remained in the presence of 1 μM TRAM-34 in 6/14 microglia. All subsequent experiments used NS309 (500 nM), which has an EC_50_ of ~30 nM for activating KCa3.1 (Strobaek et al., 2004; Wulff et al., 2007).

(iii) LPS-treated microglia. As noted in the Introduction, KCa3.1 channels are involved in classical activation of rat microglia but 24 h LPS treatment did not alter *KCNN4* expression (Kaushal et al., 2007). The purpose of the present study was to compare unstimulated and alternative-activated microglia, and further studies will be needed to investigate classical activation evoked by other stimuli. Here, we found that at 24 h after LPS treatment, the KCa3.1 current amplitude and prevalence were not obviously changed. Relatively small KCa3.1 currents were seen in 3/9 microglia, and the mean NS309-evoked current of the responding cells was 3.6 ± 1.5 pA/pF (*n* = 3). This observation does not rule out the possibility that the current can be activated by other stimuli or at other times after LPS treatment.

#### Time course of KCa3.1 current induction and need for protein synthesis

**FIGURE 3:**
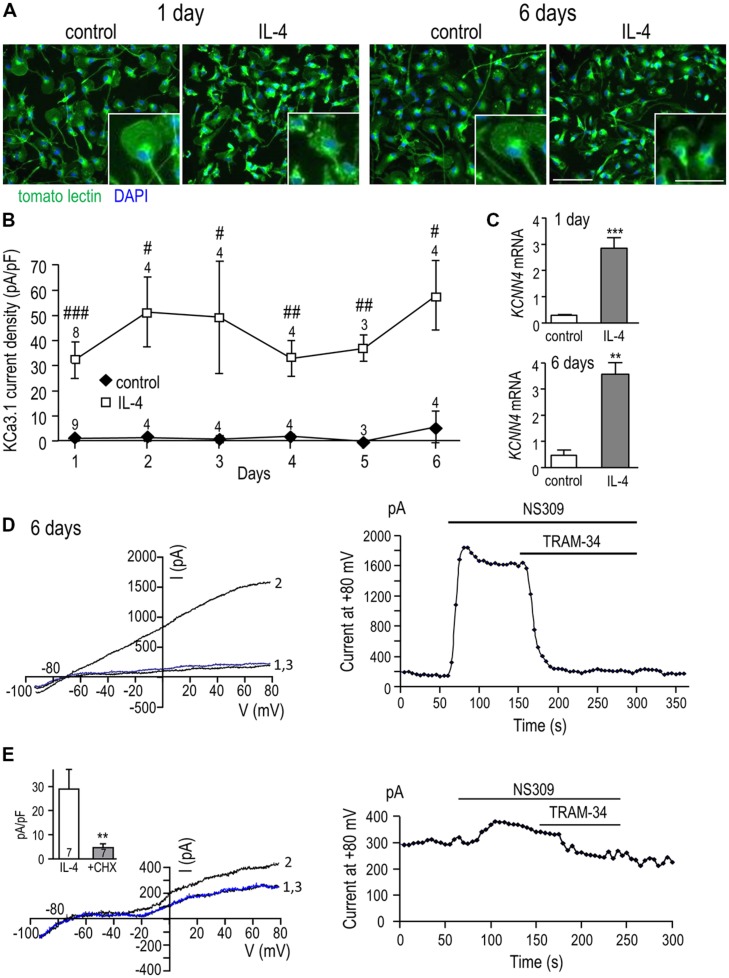
**KCa3.1 current by IL-4 remains elevated over time and requires *de novo* protein synthesis. (A)** Effect of IL-4 treatment on microglial morphology. Primary rat microglia were treated with 20 ng/ml IL-4, fixed 1 or 6 days later, and stained with tomato lectin (green) to identify microglia and DAPI (blue) to label nuclei. Scale bars = 100 μm (main images) and 50 μm (insets). **(B)** Microglia were treated with 20 ng/ml IL-4, and patch clamp experiments were performed from 1 to 6 days later. In all experiments, KCa3.1 currents were evoked by NS309 and blocked with TRAM-34. TRAM-34-sensitive KCa3.1 current densities (pA/pF; mean ± SEM for the number of cells indicated) were compared at each time point in control (unstimulated) microglia versus IL-4 treated cells. [Note: For unstimulated cells, error bars were often smaller than the symbol.] Statistical differences at each time point were determined using unpaired *t*-tests, and indicated as ^#^*p* < 0.05, ^##^*p* < 0.01, ^###^*p* < 0.001. **(C)** Quantitative real-time qRT-PCR showed a 10-fold increase in *KCNN4* transcript levels at 1 day and an eightfold increase at 6 days following IL-4 treatment compared with unstimulated cells. Values are expressed as mean ± SEM. The number of individual cultures was 6 each for 1 day control and IL-4, 3 for 6 day control and 5 for 6 day IL-4. An unpaired *t*-test was used to determine differences between unstimulated and IL-4-treated microglia: **p < 0.01, ***p < 0.001. **(D)** A representative recording at 6 days after IL-4 treatment shows currents evoked by voltage ramps from – 100 to +80 mV (holding potential, –70 mV) with control bath solution (trace “1”), after adding NS309 (trace “2”), and 1 μM TRAM-34 (trace “3”). The time-course shows current activation (measured at +80 mV) and its block by 1 μM TRAM-34. **(E)** To assess the need for protein synthesis, microglia were cultured for 24 h with 20 ng/ml IL-4 with or without 10 nM cycloheximide (CHX). The representative recording (same protocol as **D**) shows a cycloheximide-treated cell (left panel) with control bath solution (trace “1”), after adding NS309 (trace “2”), and 1 μM TRAM-34 (trace “3”), and the time course (right panel) measured at +80 mV. *Inset:* Summary of the TRAM-34-sensitive KCa3.1 current density for microglia treated with IL-4 alone or with IL-4+CHX. Statistical differences were determined by an unpaired *t*-test; ^##^*p* < 0.01.

We recently showed that a unipolar morphology with a large lamellum at the leading edge and a trailing uropod is characteristic of migrating rat microglia (Siddiqui et al., 2012; Vincent et al., 2012), and found that IL-4 treatment increases their migratory capacity (Lively and Schlichter, 2013). Here, we examined the microglia morphology at 1 and 6 days with and without IL-4 treatment and quantified the KCa3.1 current over the first 6 days after IL-4 (**Figure 3**). At 1 and 6 days, most unstimulated microglia were unipolar with a lamellum and uropod that are evident in the higher-magnification insets; the remainder were bipolar. Similarly, at both times, most IL-4-treated microglia were unipolar with a lamellum and uropod, although the lamellum was often smaller and more ruﬄed than in unstimulated cells. Thus, cell size and morphology were not indicators of the changes in *KCNN4* expression and KCa3.1 current in the alternative-activation state. For consistency in quantifying the KCa3.1 current over time, patch-clamp recordings were conducted on unipolar microglia with a distinct lamellum and a uropod, and all currents were normalized to the cell size (capacitance in pF). As summarized in **Figure 3B**, robust TRAM-34-sensitive KCa3.1 currents were reliably evoked by NS309 in IL-4-treated cells, and were present on all days tested (1-6 days). This induction of current was paralleled by increases in *KCNN4* expression, which increased ~10-fold at 1 day and ~8-fold at 6 days (**Figure 3C**). At 1 day (**Figure 2D**) and 6 days after IL-4 treatment (**Figure 3D**), the NS309-evoked current was substantially blocked by TRAM-34. We found that *de novo* synthesis of KCa3.1 protein was required for induction of the KCa3.1 current. That is, in microglia that were treated for 24 h with IL-4 and the protein synthesis inhibitor, cycloheximide, the current was 84% smaller (4.7 ± 1.5 pA/pF) than in IL-4-treated control cells (29.0 ± 8.1 pA/pF; **Figure 3E**).

### UP-REGULATION OF *KCNN4* AND KCa3.1 CURRENT IS MEDIATED BY THE TYPE I IL-4 RECEPTOR, AND REQUIRES Ras/MEK/ERK AND AP-1 SIGNALING

**FIGURE 4:**
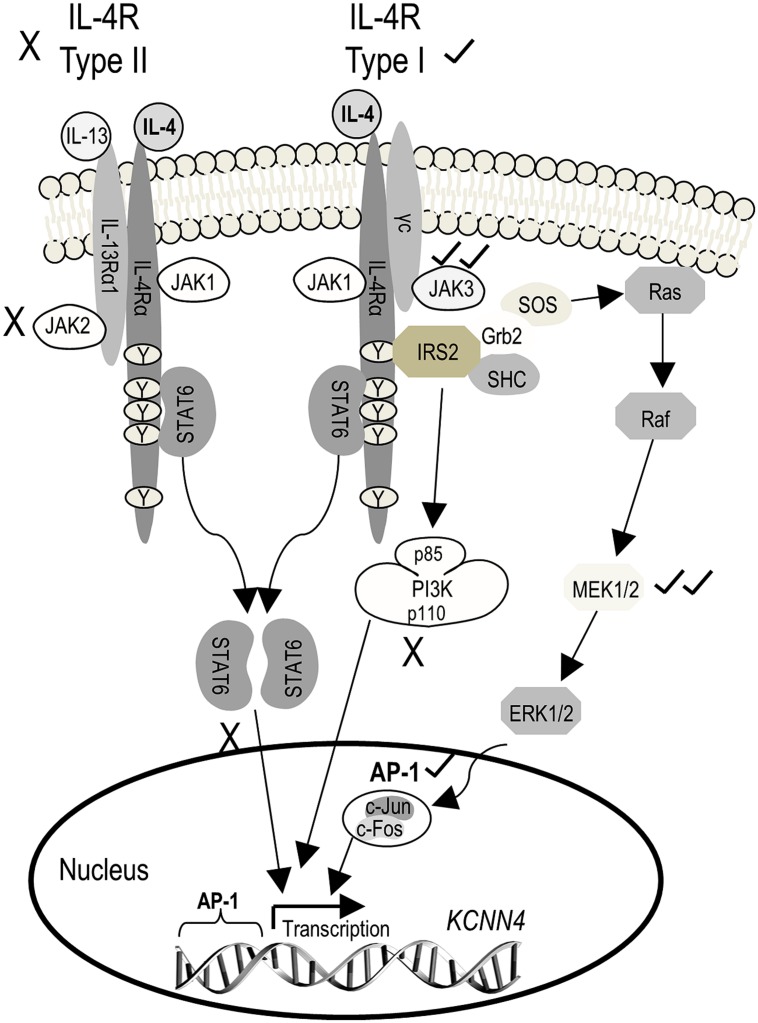
**Schematic diagram depicting known IL-4 signaling pathways and the outcomes observed.** The initial step is IL-4 binding to the IL-4 receptor α subunit (IL-4Rα), which can then heterodimerize with either the common gamma chain (γC) to form the type I receptor or with IL-13Rα1 (which binds IL-13) to form the type II receptor. Heterodimerization of either receptor subtype activates JAKs but the specific JAK depends on the receptor subunit composition. JAK1 associates with IL-4Rα, and is thus common to both receptor subtypes. JAK3 associates with γC of the type I receptor, and JAK2 associates with IL-13Rα1 of the type II receptor. For both receptor subtypes, activated JAKs phosphorylate tyrosine residues on IL-4Rα, which then act as docking sites for signaling molecules (i.e., STAT6, IRS2) that are then phosphorylated. Phosphorylated STAT6 dimerizes and translocates to the nucleus where it acts as a transcription factor, binding to the promoter of IL-4 (and IL-13) responsive genes. Recruitment of IRS2 is specific to the type I receptor, and activates downstream PI3K and Grb2 signal cascades which, like STAT6, change gene expression through the transcription factor, AP-1. (For more details on IL-4 signal transduction, see Kelly-Welch et al., 2003; Zamorano et al., 2003; Oh et al., 2010.) AP-1, activator protein-1; Grb2, growth factor receptor-bound protein 2; IL-4, interleukin 4; IL-13, interleukin-13; IRS2, insulin receptor substrate 2; JAK, Janus kinase; PI3K, phosphatidylinositol 3-kinase; STAT6, signal transducer and activator of transcription 6. *Inhibitors used:* 1 μM SR11302 for AP-1; 10 μM AG490 for JAK2/3; 30 nM TG101348 for JAK2; 10 nM tofacitinib for JAK3; 20 μM PD098059 or 10 μM U0126 for MEK1/2; 100 nM wortmannin for PI3K; 200 nM AS1517499 for STAT6.

The next goal was to determine which IL-4 receptor and signaling pathway was responsible for the increase in *KCNN4* mRNA and KCa3.1 current. To simplify the explanation of the experiments and results, **Figure 4** shows known signaling pathways downstream of type I and II IL-4 receptors and the molecules we found to affect *KCNN4* expression and KCa3.1 current. [Note: The figure legend defines the signaling molecules, inhibitor names and their targets.] Type II receptors interact with JAK1 and JAK2, and signal through STAT6 only. Type I receptors interact with JAK1 and JAK3, and their downstream signaling is usually through two pathways; i.e., one mediated by STAT6, and one by IRS2 and PI3K (Heller et al., 2008). Less commonly observed is that IRS2 can recruit the Grb2 adaptor protein, which associates with SOS and activates downstream Ras/MAP kinase pathways (p38, JNK, ERK1/2; Jiang et al., 2000; Wills-Karp and Finkelman, 2008). While use of the latter pathway apparently depends on cell type, IL-4 does activate it in keratinocytes (Wery-Zennaro et al., 2000) and in T- and pro-B-lymphocyte cell lines (Hunt et al., 2002; Canfield et al., 2005). Activated ERK1/2 then translocates to the nucleus and can activate the transcription factor, AP-1 (c-Fos/c-Jun heterodimer; Park and Levitt, 1993; Marais and Marshall, 1996). We used inhibitors of JAK2 and JAK3 to parse out the receptor type, and inhibitors of STAT6, PI3K, MEK, and AP-1 to assess downstream pathways. Their effects on expression of *KCNN4* mRNA and KCa3.1 current will next be described in detail.

#### Janus kinases: JAK2 and JAK3

**FIGURE 5:**
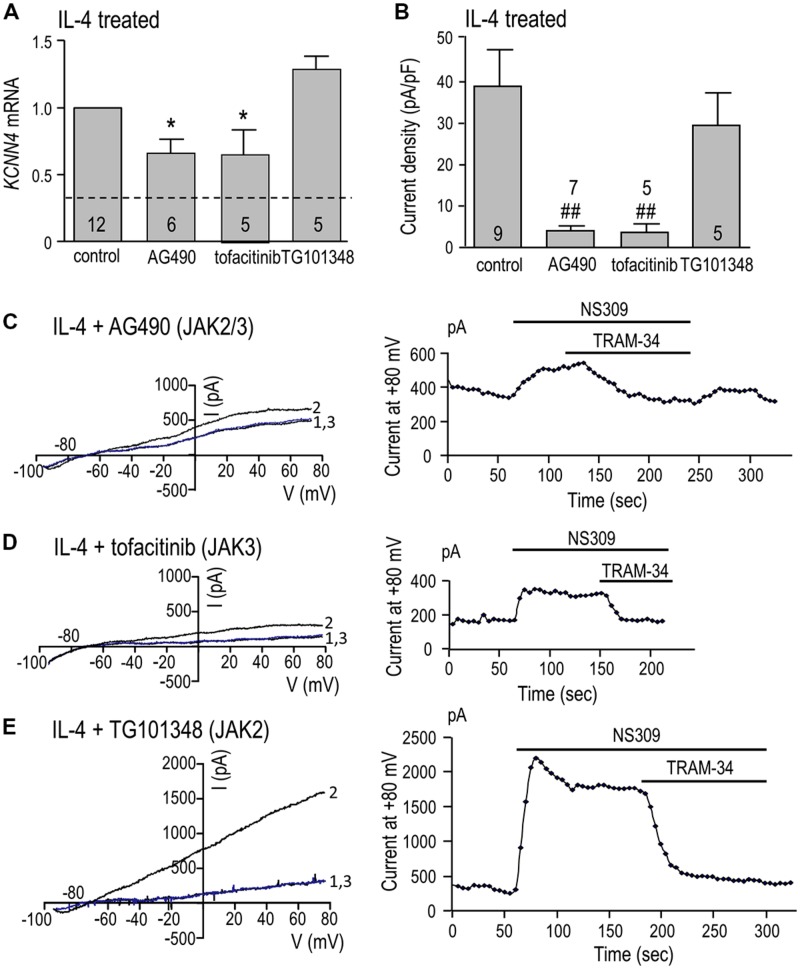
**JAK3 is required for induction of *KCNN4* mRNA and KCa3.1 current in IL-4 treated microglia.** Rat microglia were treated for 24 h with 20 ng/ml rat recombinant IL-4 without (control) or with a JAK inhibitor: 10 μM AG490 (for JAK2/3), 10 nM tofacitinib (for JAK3), or 30 nM TG101348 (for JAK2). **(A)** IL-4-mediated induction of *KCNN4* mRNA was examined using quantitative real-time qRT-PCR. Control *KCNN4* mRNA levels in unstimulated microglia (no IL-4) are indicated by the dashed line. *KCNN4* levels were normalized to IL-4-treated cells (set to 1.0) and compared with and without each inhibitor. **(B)** Summary of TRAM-34-sensitive KCa3.1 current densities (pA/pF). NS309-evoked currents were measured at +80 mV in IL-4-treated microglia, with or without a JAK inhibitor. In **A**,**B**, values are mean ± SEM for the number of cultures or cells indicated, and differences from control cells were determined using a 1-way ANOVA with Tukey’s *post hoc* test: 1 symbol indicates *p* < 0.05; two symbols, *p* < 0.01. **(C-E)** Representative current activation (left panels) and time courses (right panels) for three microglial cells; each treated with a different JAK inhibitor. Currents were elicited by repeated voltage ramps from –100 to +80 mV from a holding potential of –70 mV, and each example shows superimposed currents in normal bath solution (“1”), after bath application of 500 nM NS309 (“2”), and 1 μM TRAM-34 in the continued presence of NS309 (blue trace, “3”).

*KCNN4* expression (**Figure 5A**) and KCa3.1 current density (**Figure 5B**) were quantified in cells treated for 24 h with IL-4 with or without a JAK inhibitor. *KCNN4* expression was reduced 34% by the JAK2/3 inhibitor, AG490. This regulation is due to JAK3 because the selective JAK3 inhibitor, tofacitinib, reduced the transcript level by 36%, while the selective JAK2 inhibitor, TG101348, did not. [The possible increase in *KCNN4* by TG101348 did not reach statistical significance.] The same pattern of drug sensitivity was seen for the KCa3.1 current but the effects were greater. The summarized current densities (**Figure 5B**) and representative KCa3.1 current traces and time-courses (**Figures 5C–E**) show that AG490 reduced the current density by 90% (from 38.9 ± 8.3 to 4.0 ± 1.0 pA/pF), tofacitinib reduced it by 92% (to 3.7 ± 1.7 pA/pF), and with TG101348, the current remained at 29.5 ± 6.9 pA/pF. By implicating JAK3, these results show that the type I IL-4 receptor is involved in up-regulating *KCNN4* mRNA and KCa3.1 current.

#### STAT6, PI3K, MEK, and AP-1

**FIGURE 6:**
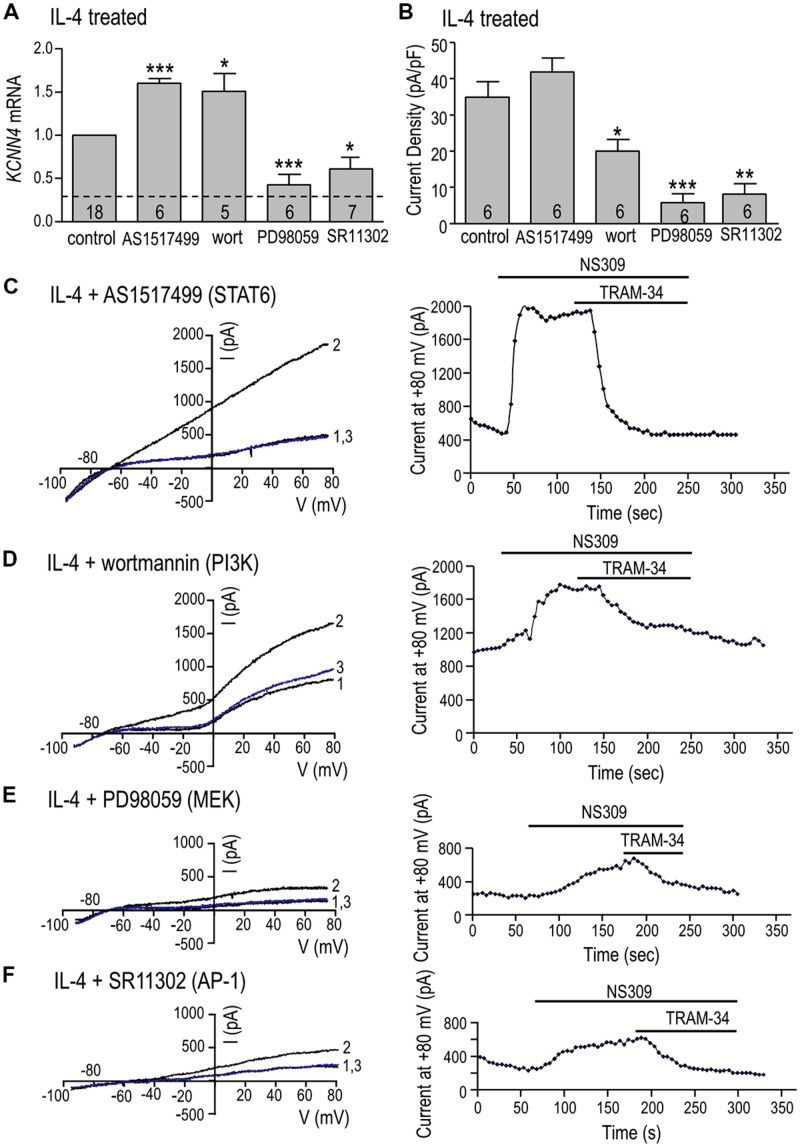
**Ras/MEK/ERK and AP-1 signaling are required for induction of *KCNN4* mRNA and KCa3.1 current in IL-4 treated microglia. (A–F)** Rat microglia were treated for 24 h with 20 ng/ml rat recombinant IL-4, without (control) or with an inhibitor of a signaling molecule: 200 nM AS1517499 (for STAT6), 100 nM wortmannin (for PI3K), 20 μM PD098059 (for MEK1/2), or 1 μM SR11302 (for AP-1). **(A)** IL-4-mediated induction of *KCNN4* mRNA was examined using quantitative real-time qRT-PCR. Control *KCNN4* mRNA levels in unstimulated microglia (no IL-4) are indicated by the dashed line. *KCNN4* levels were normalized to IL-4-treated cells (set to 1.0) and compared with and without each inhibitor. **(B)** Summary of TRAM-34-sensitive KCa3.1 current densities (pA/pF) that were evoked by NS309 and measured at +80 mV in IL-4-treated microglia with or without an inhibitor. In **A**,**B**, values are mean ± SEM for the number of cultures or cells indicated, and differences from control cells were determined using a 1-way ANOVA with Tukey’s *post hoc* test: 1 symbol indicates *p* < 0.05; two symbols, *p* < 0.01; three symbols, *p* < 0.001. **(C–F)** Representative current activation (left panels) and time courses (right panels) for four microglial cells; each treated with a different inhibitor. Currents were elicited by repeated voltage ramps from –100 to +80 mV from a holding potential of –70 mV, and each example shows superimposed currents in normal bath solution (“1”), after bath application of 500 nM NS309 (“2”), and 1 μM TRAM-34 in the continued presence of NS309 (blue trace, “3”).

*KCNN4* expression (**Figure 6A**) and KCa3.1 current density (**Figure 6B**) were quantified after IL-4 treatment, with or without a STAT6 inhibitor (AS1517499), PI3K inhibitor (wortmannin), MEK inhibitor (PD98059) or AP-1 inhibitor (SR11302). We had anticipated that *KCNN4* induction would require STAT6 or PI3K but surprisingly, inhibiting either one increased *KCNN4* in IL-4-treated cells: by 60% for AS1517499 and 50% for wortmannin (**Figure 6A**). The MEK inhibitor, which was used to assess involvement of the Ras/Raf/MEK pathway, reduced *KCNN4* to nearly the level of unstimulated microglia (without IL-4). Consistent with a role for the MEK/ERK pathway, inhibiting the transcription factor, AP-1, reduced *KCNN4* expression by 40%. There were similarities and differences in effects of these inhibitors on the KCa3.1 current in IL-4-treated microglia. Summarized current densities (**Figure 6B**), and representative current traces and time courses (**Figures 6C–F**), show that the MEK inhibitor reduced the current by 83% (to 5.8 ± 2.3 pA/pF) but the STAT6 inhibitor did not reduce it. [The MEK inhibitor, U0126, similarly reduced *KCNN4* and KCa3.1 current (data not shown)]. The AP-1 inhibitor reduced the KCa3.1 current density by 69% (from 34.9 ± 4.4 to 7.4 ± 2.5 pA/pF). Surprisingly, the PI3K inhibitor reduced the current by 43%; from 34.9 ± 4.4 to 20.0 ± 3.2 pA/pF. Together, these results suggest that the elevated *KCNN4* expression and KCa3.1 current in alternatively activated microglia require Ras/MEK/ERK signaling to AP-1, while the channel function also requires PI3K.

### MICROGLIAL MIGRATION IS INCREASED BY IL-4, AND REQUIRES KCa3.1 ACTIVITY, JAK3, Ras/MEK/ERK, AND AP-1 SIGNALING

**FIGURE 7:**
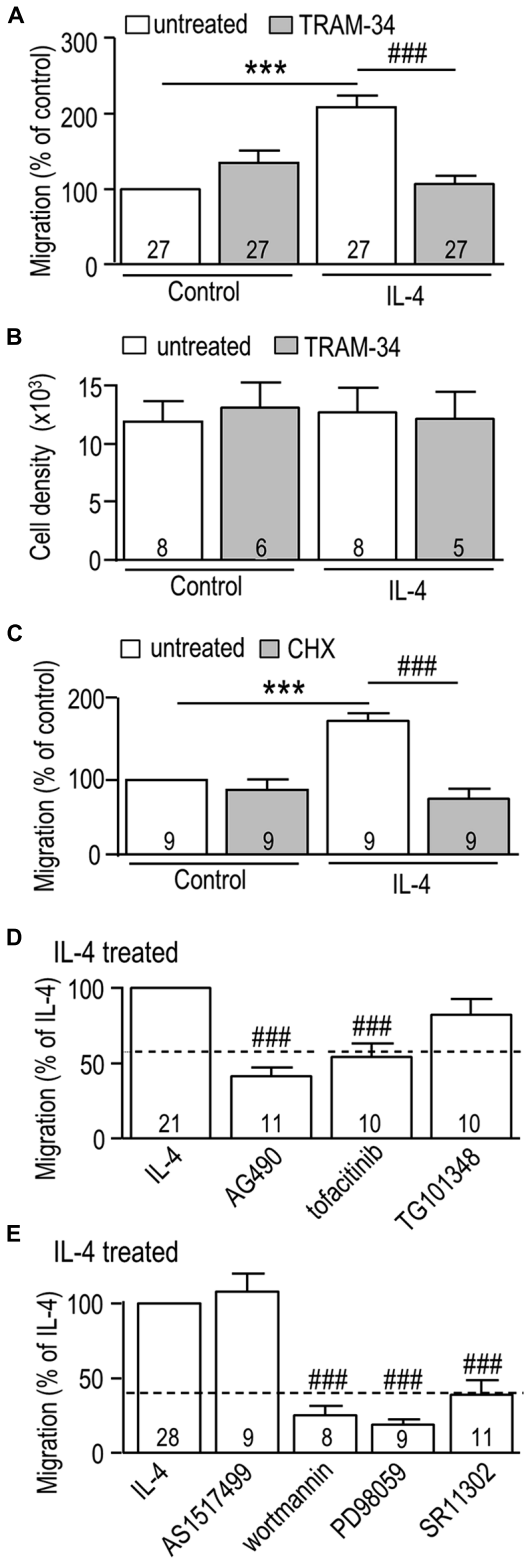
**The IL-4 induced increase in microglial migration is mediated through JAK3 and MEK signaling, and requires AP-1 activation, new protein synthesis, and KCa3.1 channel function. (A,C–E)** Rat microglia were seeded in Transwell^TM^ chambers and left unstimulated (control cells) or treated for 24 h with 20 ng/ml rat recombinant IL-4. When used, each inhibitor was added immediately before adding IL-4. Cells were fixed after 24 h, and microglia that had migrated to the underside of each filter were counted in five random fields. **(B)** Cell proliferation was analyzed using a CyQuant proliferation assay. Treatments were: **(A,B)** the KCa3.1 channel blocker, 1 μM TRAM-34; **(C)** the protein synthesis inhibitor, 10 nM cycloheximide (CHX); **(D)** 10 μM AG490 (JAK2/3 inhibitor), 10 nM tofacitinib (JAK3 inhibitor), 30 nM TG101348 (JAK2 inhibitor); **(E)** 200 nM AS1517499 (STAT6 inhibitor), 100 nM wortmannin (PI3K inhibitor), 20 μM PD098059 (MEK1/2 inhibitor), 1 μM SR11302 (AP-1 inhibitor). Data are expressed as mean ± SEM, with the numbers of individual cultures indicated. Note that a separate set of control cultures was used for each experiment (control values indicated by dashed lines in **D,E**). A 2-way ANOVA with Bonferroni’s *post hoc* test (**A–C**) or a 1-way ANOVA with Tukey’s *post hoc* test **(D,E)** revealed differences (*p* < 0.001) between unstimulated and IL-4-treated microglia (***) and the effects of each antagonist (###).

We first corroborated our recent finding (Lively and Schlichter, 2013) that LPS decreases and IL-4 increases the migratory capacity of rat primary microglia, and then we tested whether KCa3.1 is involved. The migration of LPS-treated cells was 42% ± 4% (*n* = 3) that of unstimulated cells and was unaffected by TRAM-34 (42% ± 4% of the unstimulated value; *n* = 3; not illustrated). Then, based on the IL-4-evoked increases in *KCNN4* mRNA (**Figure 1C**) and KCa3.1 current (**Figure 2**), we asked whether TRAM-34 affects the IL-4-mediated increase in migration. TRAM-34 did not inhibit migration of control (unstimulated) microglia, but fully inhibited the increased migratory capacity of IL-4-treated cells (**Figure 7A**). IL-4 can act as a mitogen (reviewed in Wills-Karp and Finkelman, 2008), so it is important that the total cell density was not affected by IL-4 (or TRAM-34) over the 24 h test period (**Figure 7B**). As for the increase in KCa3.1 current (**Figure 3E**), protein synthesis was necessary for the enhanced migration; cycloheximide abolished the increase in IL-4-treated cells but had no effect on unstimulated microglia (**Figure 7C**). JAK3 was involved in the IL-4-mediated increase in migration; it was abolished by the JAK2/3 inhibitor (AG490) and the JAK3 inhibitor (tofacitinib), while the JAK2 inhibitor (TG101348) was ineffective (**Figure 7D**). The IL-4 mediated increase in migration was abolished by the PI3K inhibitor (wortmannin), the MEK inhibitor (PD98059), and the AP-1 inhibitor (SR11302), but not affected by the STAT6 inhibitor (AS1517499; **Figure 7E**). [An 82% reduction was also seen with the MEK inhibitor, U0126 (not shown).] Together, our results show that the same signaling pathway (JAK3, PI3K, Ras/MEK/ERK, AP-1) was involved in the IL-4-induced increase in KCa3.1 current and migration.

## DISCUSSION

Initially, it was thought that KCa3.1 channels were absent from the CNS. However, *KCNN4* transcripts or KCa3.1 protein have recently been found in microglia (see below), oligodendrocytes, reactive astrocytes (Bouhy et al., 2011), and some CNS neurons (Kaushal et al., 2007; Bouhy et al., 2011; Engbers et al., 2012). Importantly, KCa3.1 blockers have improved the outcome in several rodent models of CNS damage. For instance, edema, intracranial pressure, and lesion volume were reduced in a rat model of traumatic brain injury (Mauler et al., 2004), as was disability and pro-inflammatory chemokine and cytokine expression in the spinal cord in a murine model of multiple sclerosis (Reich et al., 2005). We reported that the KCa3.1 blocker, TRAM-34, reduced neurodegeneration following optic nerve damage (Kaushal et al., 2007), and reduced the loss of tissue and neurons, and locomotor impairment after spinal cord injury in the mouse (Bouhy et al., 2011). In a rat model of transient ischemia, TRAM-34 reduced the number of activated microglia/macrophages, reduced the infarct size, increased neuron survival, and improved the neurological outcome (Chen et al., 2011). The latter study showed increased KCa3.1 in activated microglia/macrophages at the site of injury. Together, these *in vivo* studies suggest that block of KCa3.1 in microglia reduces neurotoxicity by inhibiting the pro-inflammatory state, and they support the therapeutic development of KCa3.1 blockers for CNS disorders that involve excessive inflammation. It is crucial to understand KCa3.1 contributions to microglial functions in other activation states.

Roles of KCa3.1 have previously been addressed using cultured microglia and cell lines *in vitro*. Earlier studies with the blockers, clotrimazole and charybdotoxin, showed inhibition of superoxide production in primary rat microglia (Khanna et al., 2001), lysophosphatidic acid-induced migration of BV-2 cells (murine microglia cell line; Schilling et al., 2004b), and lysophosphatidylcholine-induced secretion of IL-1β from primary murine microglia and BV-2 cells (Stock et al., 2006). However, these blockers are not perfectly selective for KCa3.1; e.g., charybdotoxin blocks other K^+^ channels, including Kv1.3 and large-conductance Ca^2+^-activated (BK) channels that can contribute to microglial functions. Kv1.3 contributes to proliferation, the respiratory burst, and neurotoxicity of rat microglia (Schlichter et al., 1996; Kotecha and Schlichter, 1999; Khanna et al., 2001; Fordyce et al., 2005), and BK contributes to microglia-mediated neuropathic pain (Hayashi et al., 2011). More recent studies often exploit TRAM-34, which is KCa3.1 selective at 1 μM (Wulff and Zhorov, 2008). In rat microglia, TRAM-34 reduced UTP-stimulated migration (Ferreira and Schlichter, 2013), and the p38 MAPK activation, production of nitric oxide, and neurotoxicity that were evoked by lipopolysaccharide-induced classical activation (Kaushal et al., 2007). In primary murine microglia, TRAM-34 reduced neurotoxicity induced by A*β* oligomers (Maezawa et al., 2011), and their chemotactic activity and phagocytosis in response to glioblastoma-conditioned medium (D’Alessandro et al., 2013). These studies focused on pro-inflammatory (classical) activation but migration, for example, is also important in the healthy CNS. Here, we provide the first evidence that KCa3.1 contributes to the enhanced migratory capacity of alternative-activated microglia.

Supporting the pharmacological evidence for roles of KCa3.1 channels in rodent microglia, *KCNN4* transcripts are expressed in primary microglia from rats (Khanna et al., 2001; Kaushal et al., 2007) and mice (Bouhy et al., 2011; Dolga et al., 2012), and KCa3.1 protein is present in activated microglia/macrophages *in vivo* (Kaushal et al., 2007; Chen et al., 2011). Surprisingly, the presence of a KCa3.1 current has been rarely reported for primary microglial cells. In comparing our results with the literature, we will consider whether this discrepancy reflects the microglial activation state, differences between primary cells and cell lines, experimental differences or the species. (i) Alternative activation is thought to shift microglia from a pro-inflammatory state to a resolving state that facilitates repair (Sica and Mantovani, 2012). Our results suggest that KCa3.1 is expressed in microglia in diverse activation states. Previously, we only occasionally detected a small-amplitude KCa3.1 current in unstimulated rat microglia (Khanna et al., 2001; Kaushal et al., 2007). Here, we detected a KCa3.1 current in 6/19 unstimulated rat microglia but the amplitude was small (0.7–1.7 pA/pF). These cells were similar to resting neonatal microglia in being highly migratory (Lively and Schlichter, 2013) and phagocytic (Sivagnanam et al., 2010), and were similar to resting adult microglia in having very low expression of numerous activation markers. These included low expression of the classical-activation markers, activated p38 MAPK and NFκB (Kaushal et al., 2007; Schlichter et al., 2010), IL-1β, TNFα, iNOS, IL-10, BDNF (Sivagnanam et al., 2010; Liu et al., 2013; Lively and Schlichter, 2013), and the alternative activation markers, MRC1, Arg1, and CD163 (Liu et al., 2013; Lively and Schlichter, 2013). We previously found that *KCNN4* mRNA was not affected in classical-activated primary microglia from rats (Kaushal et al., 2007) and mice (Bouhy et al., 2011) but we did not examine the currents. We now show that with IL-4-mediated alternative activation, *KCNN4* transcripts and KCa3.1 currents were dramatically up-regulated such that 17/18 cells had a large current (15–39 pA/pF). The current was induced within 1 day and sustained until at least 6 days, which was the longest time tested. (ii) There are apparent discrepancies in reported experimental conditions required to activate the KCa3.1 current in primary microglia and cell lines. For cloned KCa3.1 channels and the native channels in most cell types, the EC_50_ value for activation by Ca^2+^ is below 1 μM (Jensen et al., 2001; Wei et al., 2005; Wulff and Zhorov, 2008). Whole-cell recordings with 1 μM intracellular Ca^2+^evoked KCa3.1 currents in the murine BV-2 microglial cell line (Schilling et al., 2002, 2004a,b; Stock et al., 2006) but not in the murine C8-B4 cell line, even when the channel activator, DC-EBIO, was present (Moussaud et al., 2009). For the rat MLS-9 microglial cell line, we routinely activate a large TRAM-34-sensitive KCa3.1 current but only with high internal Ca^2+^ (EC_50_ ~ 7 μM) or lower Ca^2+^ and a channel activator (e.g., riluzole, UTP; Ferreira and Schlichter, 2013; Liu et al., 2013). Similarly, in the present study, current activation in alternative-activated microglia required 1 μM intracellular Ca^2+^ and a KCa channel activator. Riluzole, 1-EBIO and NS309 are thought to act by increasing the Ca^2+^ sensitivity by up to an order of magnitude (Pedersen et al., 1999; Syme et al., 2000; Pedarzani et al., 2001). While mechanisms underlying the low Ca^2+^ sensitivity of the channels in rat microglia are unknown, they will be important to investigate in future and might contribute to a failure to record KCa3.1 currents in other microglia. (iii) There might be species differences in expression of KCa currents in primary microglia but this has not been directly assessed and there are also experimental differences in the studies. In whole-cell recordings with ~1 μM intracellular Ca^2+^, microglia from NMRI mice had charybdotoxin-sensitive KCa3.1-like currents of ~200 to >1000 pA (Eder et al., 1997; Schilling et al., 2002) but their prevalence and mean amplitude were not reported. The microglial activation state was not determined; however, the cells had been cultured with macrophage colony stimulating factor for 1–2 weeks, and astrocyte-conditioned medium for a few days. In primary microglia from C57BL6 mice, a TRAM-34-sensitive KCa3.1 current was activated in 11/17 cells, and was 50–100 pA in the recordings shown (Maezawa et al., 2011). Most *in vitro* studies use cultured neonatal microglia, and there is some indication of differences in KCa currents related to cell culturing and animal age. No KCa3.1 currents were seen in microglia in acute brain slices from juvenile mice but a BK current was reported after the slices were cultured (Schilling and Eder, 2007). Discrepancies in reports of BK currents further support the possibility that both KCa currents are species-dependent or adversely affected by experimental conditions. For instance, a BK current was not seen in rat microglia, even after damage caused by facial nerve axotomy (Boucsein et al., 2000); whereas, it was present in acute brain slices from mice (Menteyne et al., 2009; Hayashi et al., 2011), and human epileptic patients (Bordey and Spencer, 2003). Surprisingly, in the latter two studies BK was activated without elevating intracellular Ca^2+^. Together, these results highlight the need for future studies to directly compare expression and roles of KCa currents in primary microglia of different species under identical experimental conditions.

In addressing the signaling pathways responsible for increasing *KCNN4* expression and KCa3.1 currents in microglia, our results have broader implications for cells that express and use KCa3.1 or IL-4 signaling. Following IL-4 binding to the IL-4Rα subchain, changes in gene expression can occur through STAT6 or IRS2-PI3K pathways, and less commonly by IRS2-Grb2 and the Ras/MEK/ERK pathway (see **Figure 4**). There is emerging evidence that Ras/MEK/ERK signaling is important for alternative activation of microglia (Zhou et al., 2012). Activated ERK1/2 translocates to the nucleus and activates transcription through the c-Fos/c-Jun heterodimer, AP-1 (Park and Levitt, 1993; Marais and Marshall, 1996). We found that IL-4 binding to the Type I receptor was responsible for increasing *KCNN4* mRNA and KCa3.1 current. The increase in current required protein synthesis and was mediated by JAK3, the Ras/MEK/ERK pathway and AP-1. The same receptor and signaling pathway was involved in increasing the migratory capacity and its KCa3.1 dependence in alternative-activated microglia. There is some literature on pathways regulating KCa3.1 expression but connections between the channel, IL-4 and AP-1 have not previously been addressed. Ras/MEK/ERK signaling up-regulates KCa3.1 in several cell types (Pena et al., 2000; Grgic et al., 2005, 2009; Si et al., 2006). Promoter analysis of the *KCNN4* gene identified sites for AP-1 (and Ikaros-2), and increased expression of KCa3.1 in mitogen-activated T lymphocytes is especially dependent on AP-1 (Ghanshani et al., 2000).

Two observations that warrant further study are that, in IL-4-treated microglia, inhibiting either STAT6 or PI3K increased *KCNN4* mRNA expression, but inhibiting PI3K decreased the current. While speculative, two possibilities seem reasonable. (i) PI3K can exert post-translational regulation of KCa3.1 current; i.e., inhibiting PI3K with wortmannin reduced the current from cloned human KCa3.1 channels expressed in CHO cells, and native channels in activated human CD24^+^ T lymphocytes (Srivastava et al., 2006). (ii) Transcription regulation might be subject to negative feedback, because potential cross-talk mechanisms exist in IL-4 receptor signaling pathways (reviewed in Nelms et al., 1999). JAK1-3 molecules can be inhibited by their interaction with SH2-domain proteins following STAT6 activation; for example, by cytokine-induced SH2 (CIS) and suppressor of cytokine signaling 3 (SOCS-3). After IL-4 receptor engagement, Ras can be inactivated by recruitment of a Ras GTPase activating protein by the p62^dok^ family protein, FRIP (IL-4 receptor interacting protein). If either JAK3 or Ras is inhibited, reduced *KCNN4* expression is anticipated.

Microglial activation is multi-faceted, and will depend on the type of stimulus, time after stimulation and factors in the local milieu (Stout, 2010; Luo and Chen, 2012). Our finding that KCa3.1 expression and contributions are regulated by the microglial activation state will have implications for targeting the KCa3.1 channel to modulate CNS inflammation. The increased migratory capacity and contribution of KCa3.1 in alternative-activated microglia is intriguing. Migration is a crucial property of microglia whether in the healthy brain during development or after damage, but the consequences will depend on the function of the cells at the target site. Microglia normally migrate within the developing CNS to help shape brain architecture and, to minimize bystander damage, it would be beneficial if they are in a non-damaging state while migrating. After CNS damage, microglia migrate to injury sites and again, a non-cytotoxic state would be beneficial. If KCa3.1 blockers inhibit migration of only alternative-activated microglia, as our results suggest, they might allow these cells to remain longer at the damage site to resolve the pro-inflammatory state and promote repair. This work also suggests that the timing of applying KCa3.1 blockers after CNS damage will be important in order to allow beneficial contributions of microglia.

## AUTHOR CONTRIBUTIONS

Lyanne C. Schlichter, Starlee Lively, and Roger Ferreira contributed to the conception and design of this study. Starlee Lively performed the NanoString analysis, cell proliferation, staining and migration assays. Roger Ferreira conducted the patch-clamp electrophysiology experiments. Lyanne C. Schlichter, Roger Ferreira, and Starlee Lively contributed to manuscript preparation. Lyanne C. Schlichter, Starlee Lively, and Roger Ferreira agree to be accountable for all aspects of the work.

## Conflict of Interest Statement

The authors declare that the research was conducted in the absence of any commercial or financial relationships that could be construed as a potential conflict of interest.

## Footnotes

1 http://www.pmgenomics.ca

2 http://www.nanostring.com/support/ncounter/

3 http://bioinfo.ut.ee/primer3-0.4.0

4 http://www.stanford.edu/~cpatton/webmaxc/webmaxcE.htm
